# Real-Time Edge Computing for Road Surface Classification Using Multi-IMU Data and a Hybrid CNN-LSTM Classification Model

**DOI:** 10.3390/s26134078

**Published:** 2026-06-27

**Authors:** Luis A. Arce-Saenz, Luis A. Salazar-Calderón, Renato Galluzzi, Javier Izquierdo-Reyes, Rogelio Bustamante-Bello

**Affiliations:** 1 School of Engineering and Sciences, Tecnologico de Monterrey, Mexico City 14380, Mexico; a01271635@tec.mx (L.A.A.-S.); luis.sc@tec.mx (L.A.S.-C.); jizquierdo.reyes@tec.mx (J.I.-R.); rbustama@tec.mx (R.B.-B.); 2 Department of Mechanical and Aerospace Engineering, Politecnico di Torino, 10129 Turin, Italy

**Keywords:** road surface monitoring, edge computing, intelligent transportation systems, real-time processing, geospatial mapping, deep learning

## Abstract

Road quality monitoring is necessary for safety, ride comfort, and driver-assistance systems. The knowledge of road features enables preventive and corrective actions at vehicle and infrastructure levels. While deep learning models are effective for surface classification, transitioning them to real-time embedded environments requires optimization. This study deploys a model based on convolutional and long short-term memory neural networks to classify five road conditions using continuous vibration data from multiple inertial measurement units. Executed on a MicroAutoBox III Embedded PC, the system preprocesses data at vehicle speeds between 5.0 and 25.0 km/h. Compared to the offline baseline deployment, this edge-optimized architecture reduced inference latency by 88% (from 33.8 ms to 4.05 ms) while maintaining a fair weighted-average F1-score of 0.8751 in real-world, cross-platform conditions (against the offline baseline average F1-score of 0.9338). This processing time operates within the 11.6 ms limit required by the 86 Hz sensor polling rate. Additionally, geospatial mapping was able to localize structural anomalies, showing robustness to environmental lighting conditions, which frequently affect vision-based systems. This cyber-physical deployment suggests the feasibility of executing temporal deep learning real-time models. Future work will target highway-speed validation and domain adaptation to assess transferability across diverse vehicle suspensions.

## 1. Introduction

Road infrastructure, such as streets and highways, supports the economic development and mobility of populated centers. Its progressive deterioration is driven by continuous use and environmental conditions. This degradation increases the risk of road accidents, accelerates vehicle wear, and imposes costs on society and transportation agencies [1,2,3].

Pavement anomalies, such as potholes, cracks, and surface deformations, compromise road safety and require consistent monitoring strategies. Traditional assessment procedures, including visual inspections, citizen reports, or specialized equipment, present operational limitations. These constraints reduce their scalability and restrict their application for long-term monitoring operations [4].

In this context, vehicle instrumentation and artificial intelligence (AI)–based methods provide practical and scalable alternatives. While various data acquisition modalities are used to characterize road conditions, vibration-based sensing has seen increasing adoption in recent literature.

Specifically, Inertial Measurement Units (IMUs) and smartphones use built-in accelerometers and gyroscopes to record vertical vibrations. These devices act as standard tools for estimating the International Roughness Index and computing ride comfort indices [1,5,6,7,8,9]. Computer vision-based approaches are often affected by poor lighting, crossing shadows, or adverse weather conditions. In contrast, accelerometers and gyroscopes directly capture the vehicle’s dynamic physical response as it interacts with road surface irregularities [10]. To process this data, raw time-domain signals are initially subjected to filtering techniques, such as band-pass Butterworth filters. This step removes noise introduced by the engine or routine driver maneuvers. The filtered signals are then transformed into the time-frequency domain using techniques such as FFT, spectrograms, or power spectral density analysis [4,11].

This direct data acquisition enables low-cost, computationally lightweight information collection. In turn, this approach supports adaptable participatory crowdsourcing models. Within these frameworks, fleets of production vehicles or public transit networks transmit data via Vehicle-to-Infrastructure networks or fog-computing architectures [12,13]. This methodology can reduce monitoring costs and facilitate the near-real-time mapping of large-scale infrastructure conditions. However, sensor placement remains a critical factor for classification accuracy. Studies indicate that vibration responses differ depending on whether sensors are mounted on the vehicle’s sprung mass (e.g., chassis, dashboard, seats) or on the unsprung mass (e.g., near the tires or axles) [10].

Vibration-only analysis typically lacks the resolution to specify the exact size or type of damage. Addressing this limitation motivates a transition toward multisensor systems [8,10,14]. Current literature emphasizes sensor fusion techniques to mitigate these constraints. These approaches include operating multiple IMUs in parallel and jointly processing acceleration and angular velocity signals. This combined data is frequently analyzed using hybrid architectures, such as Convolutional Neural Network (CNN)-Long Short-Term Memory (LSTM) models, to improve classification accuracy. Additional methodologies incorporate microphones to capture vibroacoustic signatures [15,16]. Furthermore, dual-acquisition setups combine a smartphone’s inertial sensors with its camera to compensate for the individual weaknesses of each modality. In these configurations, computer vision classifies the anomaly, while the IMU estimates the corresponding impact severity [4,14].

Computer vision, 3D sensing, and automotive radar enable the detection of pavement deterioration and the accurate reconstruction of the surrounding environment. Instruments such as RGB-D sensors (e.g., Microsoft Kinect), cameras, and high-frequency radars directly capture the physical environment to deliver detailed spatial data. High-resolution cameras record 2D spatial features and textures. In parallel, 3D sensors such as Light Detection and Ranging (LiDAR) sensors and RGB-D cameras produce dense point clouds. These point clouds allow for the geometric estimation of defect depth and volume, including rutting and potholes [17,18]. Furthermore, automotive radars (e.g., 77 GHz and 79 GHz Frequency-Modulated Continuous-Wave) provide an alternative sensing modality. By analyzing backscattered range spectra, these radars classify surface roughness and differentiate material types. This radar-based approach remains robust against variations in lighting or weather conditions [19].

However, these technologies introduce operational challenges. These limitations primarily include hardware costs, high data volumes, and sensitivity to environmental factors. For example, illumination changes frequently degrade video-based measurements. Cameras and standard RGB-D units are susceptible to weather variations, motion blur, and shadows [20]. Furthermore, direct sunlight can saturate the infrared receivers in RGB-D devices. This saturation produces “flying pixels” and depth calculation errors [17]. While LiDAR systems provide high accuracy, their current cost restricts broad crowdsourced deployment. Additionally, these systems experience performance degradation in heavy rain, snow, or fog due to light-beam interference [1]. Finally, multimodal platforms generate large volumes of data that require corresponding computing power, bandwidth, and storage. These resource requirements complicate real-time processing and large-scale implementation [10,14,21].

For data analysis, machine learning methods demonstrate utility on inertial data through supervised approaches (e.g., random forests, support vector machines, and k-nearest neighbors) [22]. Additionally, unsupervised techniques (e.g., k-means on the power spectral density, PSD) facilitate defect detection while reducing labeling requirements [4,6]. Within deep learning, architectures such as U-Net and CNNs enable the pixel-level segmentation of damage in images. For time-series and multisensor data, combinations of CNNs with LSTMs provide effective modeling capabilities [10,14,23]. Furthermore, time-frequency representations (spectrograms) improve the discrimination between structural anomalies and expected elements, such as speed bumps. In recent literature, Transformers and conditional Generative Adversarial Networks with attention mechanisms have improved noise robustness and context modeling. These architectures help preserve classification accuracy during real-time operational scenarios [7].

Despite the predictive capabilities of current deep learning architectures, an operational gap remains regarding their practical deployment in transportation systems. Recent methodologies predominantly rely on the offline post-processing of collected inertial data to evaluate road conditions [10,11]. Alternatively, studies attempting near real-time monitoring frequently utilize smartphone-based applications or cloud-dependent computing architectures [4,23]. While these methods achieve stable classification metrics, they introduce latency, computational bottlenecks, and bandwidth dependencies. Consequently, there is a need for methodologies capable of processing and classifying continuous, high-frequency IMU data directly at the edge. To address this limitation, this research proposes an end-to-end cyber-physical architecture built upon the Robot Operating System 2 (ROS 2). Unlike standard sequential computing environments, ROS 2 provides a publish-subscribe framework that synchronizes simultaneous data streams from multiple sensors. By combining ROS 2 with a hardware-accelerated ONNX runtime, this study demonstrates a reduction in latency compared to previous frameworks. This approach transitions the evaluation process from offline analysis to local, on-board inference.

The proposed system captures continuous vibration data using three automotive-grade IMUs mounted on the vehicle’s sprung and unsprung masses. These inertial signals are dynamically normalized and processed by a previously trained hybrid 1D-CNN+LSTM model. This model classifies five distinct road conditions: even road, uneven road, manhole-pothole, patch, and speed bump. To contextualize these predictions, the system synchronizes the inference node with an on-board Global Positioning System (GPS) module, enabling continuous geospatial anomaly mapping.

This real-time deployment can provide baseline data for Intelligent Transportation Systems (ITS) and active infrastructure monitoring. By classifying the road surface at the edge, the architecture generates low-latency data that can inform subsequent interventions, ranging from driver alerts to the dynamic tuning of controllable suspensions. Therefore, to address the identified operational gaps in the literature, this research presents two primary contributions:

1. The cyber-physical development and deployment of a ROS 2-based edge architecture on a MicroAutoBox III Embedded PC. This setup enables real-time road surface classification, operating without reliance on cloud infrastructure.
2. The evaluation of computational feasibility of the pre-trained multi-IMU 1D-CNN+LSTM model. This assessment is conducted by optimizing the model into a low-latency ONNX runtime and synchronizing the inference pipeline with a GPS module to produce geolocated infrastructure maps.

The remainder of this article is organized as follows: Section 2 details the materials and methods, encompassing the in-vehicle hardware configuration, the ROS 2 edge computing framework, the real-time data synchronization pipeline, and the proposed 1D-CNN+LSTM model integration. Section 3 presents the experimental results, focusing on the model’s classification performance and the system’s edge inference latency. Section 4 provides a discussion of the findings and their implications. Finally, Section 5 concludes the paper and outlines directions for future research.

## 2. Materials and Methods

**Figure 1:**
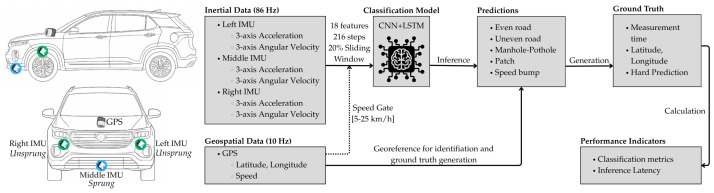
Proposed system architecture for real-time road surface anomaly detection. The framework illustrates the integration of the inertial sensors (sprung and unsprung masses) and GPS antenna, centralized on an edge-deployed 1D-CNN+LSTM model for synchronous classification and data logging.

This section details the hardware and software architecture developed for the real-time road surface monitoring system. The proposed methodology includes continuous data acquisition from in-vehicle sensors, temporal synchronization within the edge computing environment, and the deployment of a pre-trained 1D-CNN+LSTM model. The overall system framework, illustrating the data flow from the sensor layer to the classification output, is presented in Figure 1.

### 2.1. Hardware Configuration

The hardware architecture is based on the data acquisition methods established in [10,12]. However, this iteration modifies both the computing platform and the sensors to support real-time edge processing.

The system employs a MicroAutoBox III Embedded PC equipped with an Intel Xeon CPU E3-1505L v6 (2.20 GHz, 8 cores) and 16 GB of RAM, running Ubuntu 20.04.6 LTS. To establish a standalone mobile environment, the computer draws power directly from the vehicle’s 12 V electrical system. This configuration provides the computational capacity required to execute continuous data ingestion, sensor synchronization, and local 1D-CNN+LSTM model inference.

Vibration data is captured using a multi-IMU setup consisting of three WT901C 9-axis IMUs connected to the computer via a USB hub. The inertial signals are captured at a sampling frequency of 86 Hz. This frequency exceeds the typical resonance of an automotive unsprung mass (15 to 20 Hz). Consequently, this sampling rate provides the bandwidth necessary to capture the high-frequency surface interactions required by the 1D-CNN+LSTM model. These IMUs have a measuring range of ±16 g, a resolution of 0.0005 g/LSB, and an inclination accuracy of 0.2°. To record different vehicle dynamics, the sensors are placed on both the sprung and unsprung masses [12]. Two IMUs are mounted on the left and right shock absorber bottom hinges to measure the unsprung mass. The third IMU is attached to the front center of the chassis to record the inertial response of the sprung mass.

Geospatial localization and vehicle kinematics are recorded with a VK-162 USB GPS module operating at 10 Hz. This module serves two distinct purposes in the architecture. First, it provides the vehicle speed required to trigger the model inference through a conditional gate. Second, it supplies the latitude and longitude coordinates needed to geographically map the detected road anomalies.

### 2.2. ROS 2 Framework

**Figure 2:**
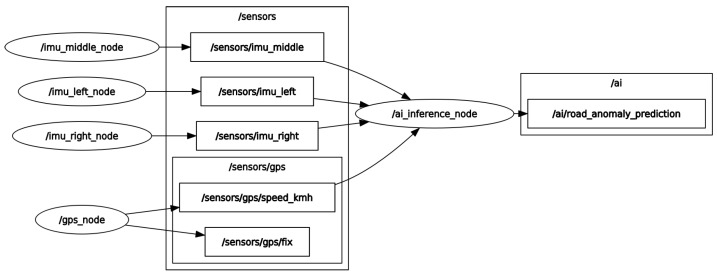
ROS 2 graph illustrating the decentralized node architecture. The hardware nodes publish raw sensor streams to dedicated topics, which are ingested asynchronously by the central inference node (/ai_inference_node) to produce the final road anomaly prediction.

The edge computing software is developed within the ROS 2 framework to synchronize asynchronous data streams from the distributed hardware. Figure 2 illustrates the decentralized node architecture. Hardware-specific nodes (/imu_left_node, /imu_middle_node, /imu_right_node, and /gps_node) operate independently. These nodes continuously publish their raw sensor streams to their dedicated topics.

The system collects 18 specific inertial features. These features comprise the three-axis acceleration and three-axis angular velocity from each sensor in the multi-IMU setup. These signals, alongside the kinematic GPS data, are aggregated at the central /ai_inference_node. This node manages data buffering and temporal alignment. Additionally, it executes the classification model inference. The classification output includes the measurement timestamp, geographic coordinates, and the predicted class. This information is published to the /ai/road_anomaly_prediction topic for system monitoring. Concurrently, a background ROSBag process records all raw sensor data and inference outputs into a .db3 file. This configuration enables real-time classification. Simultaneously, it archives the original sensor data for subsequent review and validation against the ground truth.

#### Data Synchronization

Sensor fusion requires temporal alignment across the distributed inertial streams. However, the multi-IMU setup interfaces via a centralized USB hub. Consequently, transmission delays and operating system variations prevent the simultaneous arrival of data packets. To address this asynchronous delivery, the architecture implements the ROS 2 ApproximateTimeSynchronizer. This policy functions as a message filter designed to group data streams from multiple topics that share closely aligned hardware timestamps. A rigid synchronizer is unsuitable for this application, as it would drop incoming IMU packets due to USB transmission variations. Instead, this approximate policy buffers the incoming messages. It then outputs a synchronized array if the timestamps fall within a predefined temporal tolerance.

In this system, the synchronizer is configured with a 0.025-s window. The 86 Hz sampling rate delivers a new sample approximately every 0.011 s. Therefore, this specific tolerance compensates for hardware delays while restricting the grouping to a two-frame maximum. As a result, this policy aligns the fused readings from the left, middle, and right IMUs to the corresponding physical vehicle state. This temporal alignment reduces the artificial overlap of separate road impacts.

The inference process is regulated by a speed gate to support classification accuracy. The system monitors the 10 Hz GPS speed updates. It triggers the classification model only when the vehicle operates between 5.0 and 25.0 km/h. This operational window corresponds to the speeds used to collect the training and testing data for the classification model. Restricting the system to this speed range aligns the input data with the model’s training parameters. This constraint mitigates the risk of misclassifications that may occur at highway speeds or when the vehicle is stationary.

Once gated and synchronized, the data populates a fixed-length buffer of 216 steps. This buffer holds approximately 2.5 s of vehicle movement. This array size corresponds to the required input shape for the 1D-CNN+LSTM model, which is detailed further in Section 2.3. To provide continuous detection during operation, the system employs a 20% sliding window technique. After each classification, the oldest 43 time steps are removed from the buffer. This operation advances the window while maintaining an 80% data overlap. This overlap increases the probability that sudden road anomalies are captured by the model before exiting the temporal window.

### 2.3. Deep Learning Model

The core classification engine employs a pre-trained Single-Head 1D-CNN+LSTM model initially developed and validated by [10]. A discrete classification approach was selected because inertial measurements capture the dynamic physical response of the vehicle during road interactions. Consequently, these sensors output distinct time-series patterns for specific structural anomalies, rather than the fine-grained visual details used in semantic segmentation. The five target classes (even road, uneven road, manhole-pothole, patch, and speed bump) represent prevalent road conditions in dense urban environments and produce distinct vibrational signatures. The original model was trained on an augmented, balanced dataset of time-series windows padded to 216 data points.

For this edge deployment, the model weights remain frozen. The primary objective is to minimize execution latency within the constrained computational environment of the vehicle.

Before ingestion by the neural network, the synchronized sensor data requires normalization. To minimize computational overhead on the edge device, libraries such as scikit-learn are excluded from the deployment. Instead, the 216 × 18 input tensor is normalized utilizing NumPy matrix operations. This scaling process applies a pre-calculated offset via mean subtraction and standard deviation division, expressed as:

(1) $$X_{norm}=\frac{X-\mu }{\sigma }$$

where *X* is the raw input tensor, μ is the pre-calculated mean, and σ is the standard deviation from the training dataset. This normalization aligns the real-time data with the original offline training parameters while minimizing computational latency.

The normalized data is processed sequentially by the 1D-CNN+LSTM model. First, the 1D-CNN layers extract spatial features and high-frequency patterns directly from the IMU signals. Then, these features propagate to the LSTM layers. These layers evaluate the temporal evolution of the vibrations across the 2.5-s window. This temporal analysis enables the system to distinguish between transient impacts (e.g., a manhole-pothole) and continuous textures (e.g., an uneven road). Finally, a fully connected dense layer using a softmax activation function outputs a probability distribution for the five target classes.

To achieve low-latency execution, the model was converted into the Open Neural Network Exchange (ONNX) format. Executing the model within the ONNX runtime mitigates the computational overhead associated with standard machine learning frameworks. This conversion reduces CPU execution time. This efficiency enables the processing unit to sustain the continuous classification cycle while maintaining synchronization with the high-frequency sensor inputs.

### 2.4. Experimental Setup

**Figure 3:**
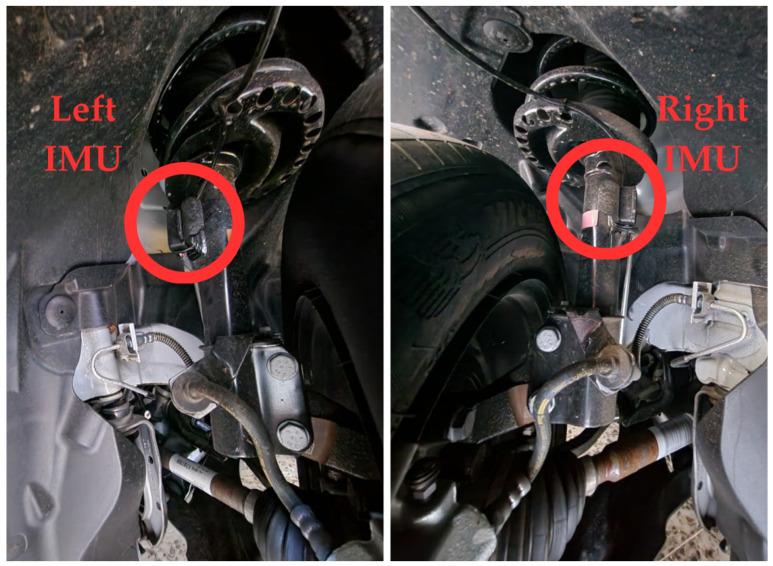
Installation of the left and right IMUs on the shock absorber bottom hinges of the test vehicle to capture unsprung mass dynamics.

To validate the edge computing system under real-world conditions, the hardware framework was installed in a 2026 Chevrolet Groove. As illustrated in Figure 3, the left and right IMUs were mounted to the shock absorber bottom hinges to measure the unsprung mass dynamics. This test vehicle differs from the platform utilized to collect the training data for the classification model [10]. Deploying the pre-trained weights on an alternative vehicle provides an initial assessment of the model’s transferability across different mechanical suspension and damping profiles.

Testing was conducted on public streets surrounding the Tecnologico de Monterrey, Mexico City Campus, in the southern region of the city. These routes were selected to provide a diverse range of road surface conditions. The driving paths included recently paved roads and degraded sections presenting surface irregularities, such as speed bumps, asphalt patches, and potholes. This environment provided the necessary road elements to evaluate all five target classes.

#### 2.4.1. Test Protocol

The testing procedure started with a 10-s stationary calibration period to record baseline inertial data. This initialization step establishes a zero-reference point for the IMUs to align the gravity vector and mitigate initial sensor bias. Following calibration, the vehicle navigated 3.75 km of urban routes, intentionally driving over the five target road surface conditions: even road, uneven road, manhole-pothole, patch, and speed bump. Throughout the testing phase, the vehicle speed was maintained between 5.0 and 25.0 km/h. This operational range satisfied the conditional speed gate, triggering the classification model and aligning the input data with the original training parameters.

#### 2.4.2. Ground Truth Generation and Synchronization

During data collection, a dashboard-mounted smartphone recorded a forward-facing video of the road surface, capturing both visual frames and geolocation data. Because the smartphone and the ROS 2 edge computer operated on independent hardware clocks, direct temporal alignment during data ingestion was not feasible.

Following data collection, the ROSBag file was parsed to extract the model inference outputs, including the measurement timestamp, geographical coordinates, predicted class, and probability distribution. These variables were exported to a CSV file for ground truth annotation.

To compensate for the temporal misalignment between the independent data streams, a post-processing methodology based on geospatial and temporal cross-referencing was employed. A custom web-based visualization tool was utilized to simultaneously render the vehicle’s classification trajectory on a map alongside the recorded video feed. Each classification was evaluated frame-by-frame by correlating the timestamp and geographic coordinates of the prediction with the corresponding video frame. To achieve temporal alignment, a static temporal offset was subtracted from the video timestamps. This offset accounts for the latency between the physical road surface interaction and the timestamp of the resulting prediction window.

**Figure 4:**
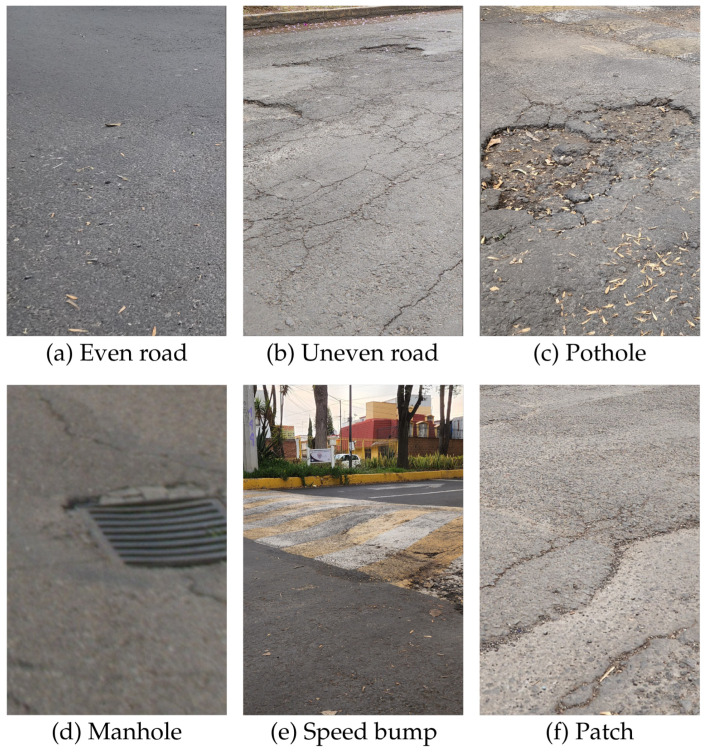
Visual representation of different road surface classification categories.

The manual annotation protocol and labeling criteria align with the methodological framework established in previous investigations [10,12,24,25,26]. Utilizing the synchronized web tool, the labeling criteria required visual confirmation that the vehicle’s front axle physically interacted with the geometric edge of the target anomaly at the corresponding geolocation of the prediction. To clarify these boundaries and the physical nature of the target conditions, Figure 4 illustrates the five target classes evaluated in this study.

The definitions for the target classes are described as follows:

- **Even Road:** Pavement sections maintaining structural integrity without surface distress. These sections generate a baseline low-amplitude dynamic response.
- **Uneven Road:** Sections exhibiting continuous surface deterioration, such as alligator cracking, rutting, or corrugation. These features produce sustained, high-frequency vehicle vibrations.
- **Manhole-Pothole:** These anomalies are grouped due to their similar sharp-edge impact profiles. Following ASTM International [27] definitions, potholes are bowl-shaped depressions resulting from localized structural failure. Manholes represent utility access points that introduce comparable negative-depth or sharp-edge surface deviations.
- **Patch:** An area of pavement replaced with new material to repair the existing surface [27]. These repairs frequently introduce material transitions and elevation inconsistencies relative to the surrounding road.
- **Speed Bump:** Traffic calming devices characterized by a predictable, low-frequency suspension stroke.

Although the frame-by-frame annotation protocol is subject to potential variance in determining the exact timestamp of impact, the 2.5-s temporal window of the classification model provides sufficient margin to accommodate these minor alignment discrepancies. This temporal overlap mitigates minor synchronization offsets while preserving classification integrity. Consequently, this manual annotation process established an independent ground truth for the 1633 physical instances captured during the test drive. The real-time predictions generated by the edge architecture were evaluated against this independent baseline, forming the basis for the performance evaluation.

## 3. Results

This section presents the validation of the proposed edge computing architecture and the 1D-CNN+LSTM model. The evaluation focuses on three key areas: computational performance on the edge hardware, classification accuracy and geospatial mapping in real-world conditions, and system robustness across varying vehicle velocities.

### 3.1. Edge Computing Performance

Processing latency exceeding the sensor polling rate results in data loss. The WT901C inertial sensors operate at 86 Hz, establishing a maximum allowable computational time of 11.6 ms per sliding window array. Therefore, quantifying system latency is necessary to validate the architecture for continuous deployment.

Benchmarking was conducted on the MicroAutoBox III Embedded PC. Execution timestamps were logged across the dataset to record two metrics. First, CPU inference latency was measured to isolate the computation time of the model. Second, total pipeline latency was measured to capture the ROS 2 execution cycle, including sensor data ingestion, temporal synchronization, dynamic normalization, model inference, and publisher overhead.

Table 1 presents the statistical distribution of the recorded latencies. The average CPU inference latency was 3.33 ms, while the total pipeline latency averaged 4.05 ms. The maximum recorded pipeline latency was 7.14 ms, which remains below the 11.6 ms threshold.

### 3.2. Classification Performance and Geospatial Mapping

The classification performance of the inference node was evaluated using a ground truth dataset of 1633 labeled samples. The dataset exhibits class imbalance, as the Even class frequency exceeds that of transient anomalies such as Patches. To quantify performance, standard metrics including accuracy, precision, recall, and the F1-score were utilized.

Accuracy measures the proportion of correct predictions across all classes, calculated as:

(2) $$Accuracy=\frac{TP+TN}{TP+TN+FP+FN}$$

where TP, TN, FP, and FN represent the true positives, true negatives, false positives, and false negatives, respectively. However, in highly imbalanced datasets, global accuracy can be deeply misleading, as the overrepresented majority class dominates the metric. Therefore, the F1-score is prioritized to assess model performance.

Precision measures the proportion of positive identifications that were actually correct:

(3) $$Precision=\frac{TP}{TP+FP}$$

where TP represents the true positives and FP denotes the false positives. Recall evaluates the proportion of actual positives that were identified correctly, defined as:

(4) $$Recall=\frac{TP}{TP+FN}$$

where FN represents the false negatives. Finally, the F1-score provides the harmonic mean of precision and recall, offering a balanced metric that is particularly robust for uneven class distributions:

(5) $$F1\text{-}Score=2\cdot \frac{Precision\cdot Recall}{Precision+Recall}$$

To summarize the overall multi-class performance, two aggregate metrics are reported. The Macro Average computes the unweighted mean of the metrics across all *N* classes, treating every class equally regardless of the sample size (support). This penalizes the 1D-CNN+LSTM model if it performs poorly on minority classes:

(6) $$Macro\;Avg=\frac{1}{N}\sum_{i=1}^{N}F1_{i}$$

In contrast, the Weighted Average calculates the mean of the metrics weighted by the support proportion ($W_{i}$) of each class, reflecting the overall system reliability based on the actual distribution of physical events encountered:

(7) $$Weighted\;Avg=\sum_{i=1}^{N}\left(F1_{i}\cdot W_{i}\right)$$

Table 2 presents the precision, recall, F1-score, and support for each target condition. To quantify the statistical variability of these metrics, 95% confidence intervals were calculated using a non-parametric bootstrapping technique with 10,000 resamples. The system achieved an overall accuracy of 0.8683 (95% CI: [0.8512–0.8849]), a macro average F1-score of 0.7756 (95% CI: [0.7477–0.8022]), and a weighted average F1-score of 0.8751 (95% CI: [0.8590–0.8905]). Class-specific F1-scores ranged from 0.4810 for the Patch class to 0.9565 for the Even road class.

**Figure 5:**
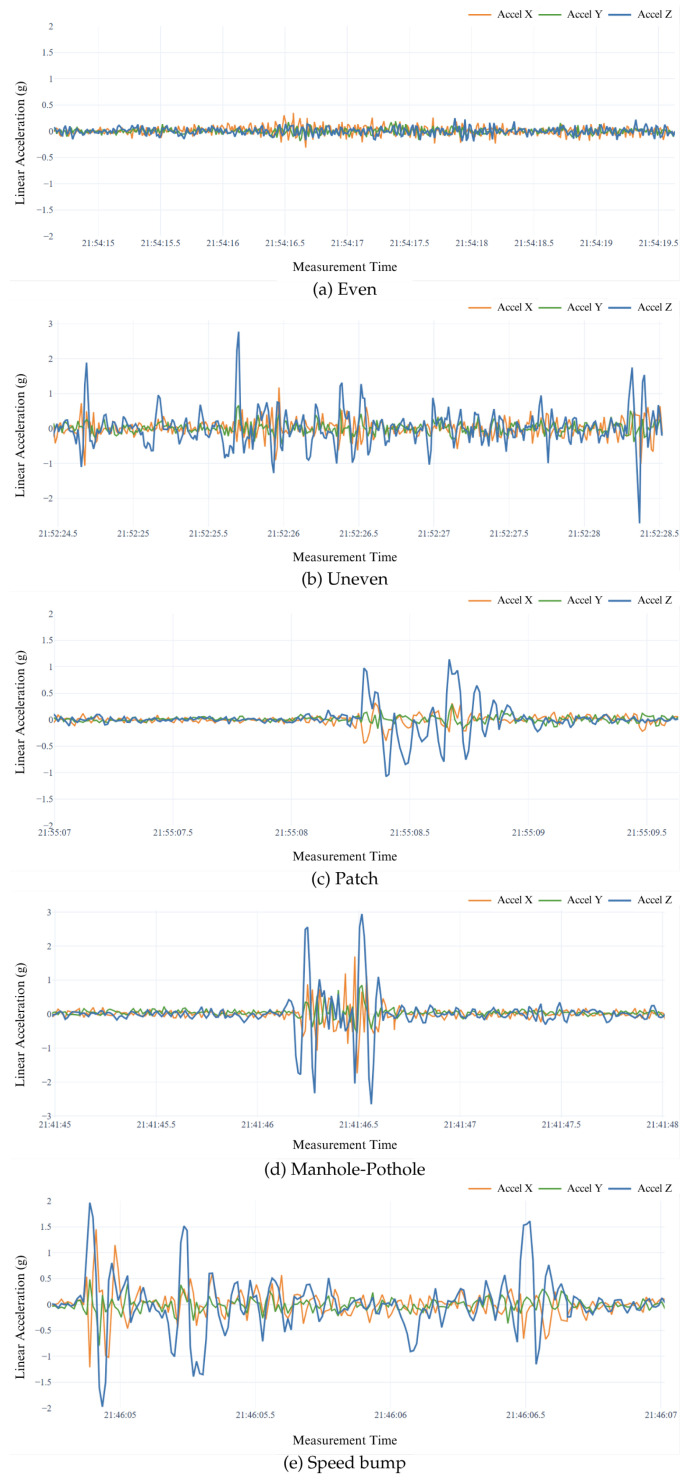
Inertial signatures (linear acceleration in g) captured by the middle IMU across the five target road conditions. The plots illustrate a representative time window for five road surface classification categories.

To visually validate the performance of the classification model, Figure 5 illustrates the inertial signatures captured across the five target classes. The time-series plots demonstrate the normalized linear acceleration over a representative measurement window. Even surfaces exhibit minimal baseline noise, approaching 0 g. In contrast, Uneven segments exhibit continuous, high-frequency vibrations, while Patches present a brief perturbation that attenuates rapidly. The Manhole-Pothole and Speed bump categories demonstrate distinct temporal profiles. Potholes display a concentrated, high-frequency response (exceeding 2.5 g), while Speed bumps present a low-frequency, dual-oscillation pattern corresponding to sequential axle impacts. These differences in temporal and amplitude features provide the physical basis for the classification performance of the 1D-CNN+LSTM model.

**Figure 6:**
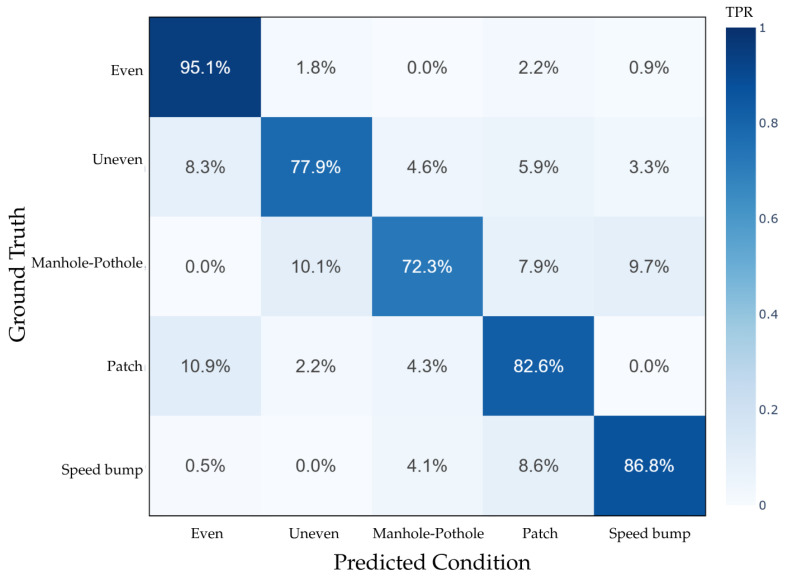
Normalized confusion matrix detailing the classification performance across the five target road surface conditions based on 1633 real-world samples.

The normalized confusion matrix (Figure 6) details the classification results, illustrating the distribution of predictions across the 1633 test instances. The Even class achieved the highest performance metrics (F1-score: 0.9565). Among the anomalies, Speed bump and Uneven surfaces produced comparable F1-scores of 0.8321 and 0.8110, respectively. The Manhole-Pothole class achieved an F1-score of 0.7975, characterized by high precision (0.8894) but lower recall (0.7228). The Patch class recorded the lowest F1-score (0.4810); while achieving a recall of 0.8261, its precision was 0.3393.

These class-specific variances demonstrate the utility of aggregate metrics. Because the 1D-CNN+LSTM model performs reliably on the most frequently encountered surface types in urban environments, it yields a weighted-average F1-score of 0.8751, indicating its operational feasibility. Conversely, the macro-average F1-score of 0.7756 provides a diagnostic baseline by highlighting the performance reduction in the underrepresented Patch class (46 samples). This discrepancy isolates the impact of deploying the model on a vehicle with different suspension kinematics without prior domain adaptation. A comprehensive discussion relating this class-by-class classification performance to standard methodologies in the literature is presented in Section 4.2.

**Figure 7:**
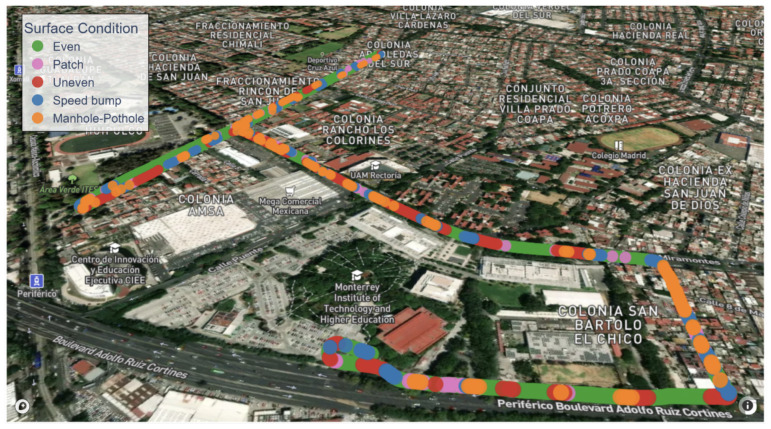
Geospatial map of the 3.75 km urban test route, plotting the continuous classification of road surface conditions generated by the real-time edge inference node.

To validate the real-world utility of the system, the output of the inference node was synchronized with the GPS coordinates to construct a geospatial map. Figure 7 presents the mapping of the 3.75 km urban test route. The route encompassed diverse scenarios, including well-maintained pavement sections with isolated speed bumps, as well as degraded sections characterized by dense surface irregularities.

**Figure 8:**
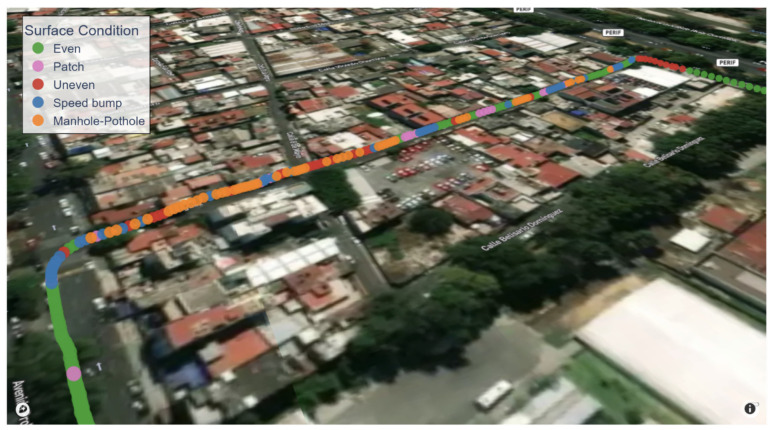
Spatial mapping of continuous anomalies along a severely degraded route section.

Spatial validation was carried out to evaluate the geographic placement of the predicted anomalies. Figure 8 shows a degraded route section, plotting multiple consecutive irregularities, such as potholes, manholes, speed bumps, and highly degraded pavement with several patches and cracks.

**Figure 9:**
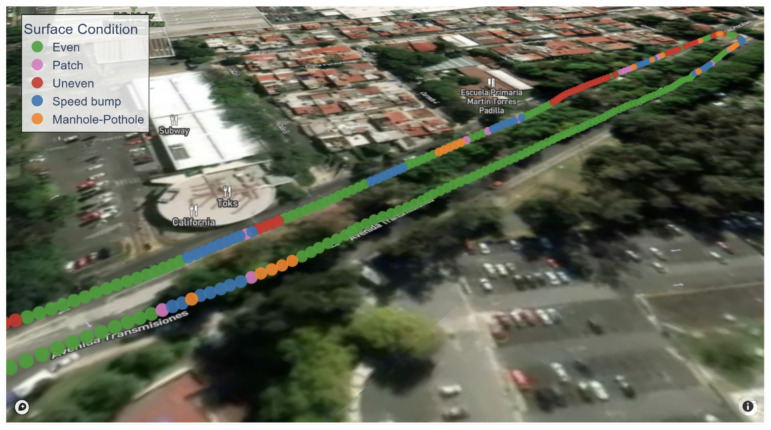
Spatial isolation of sequential speed bumps and manholes along a recently paved road surface.

In contrast, Figure 9 details a recently paved road section with no visible surface degradation. The system effectively mapped the continuous even surface, interrupted only by sequential speed bumps and specific manholes.

**Figure 10:**
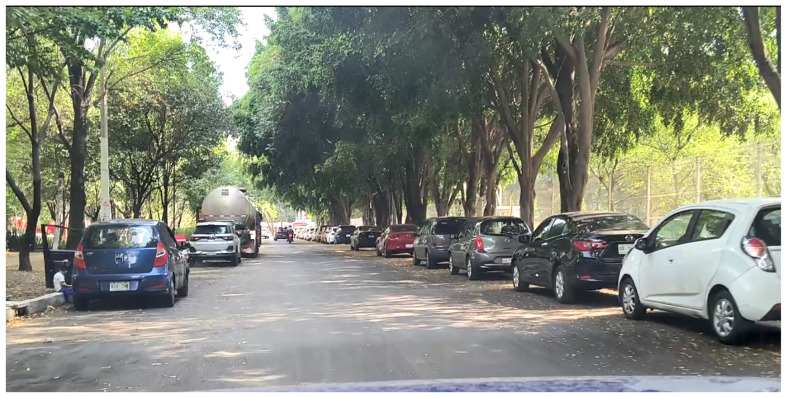
Dashboard camera view of the test route. The presence of dense, irregular tree shadows highlights the complex environmental lighting conditions present during the inertial data collection.

Figure 10 provides a simultaneous dashboard camera frame from the route shown in Figure 9. The image records dense tree shadows across the pavement. This visual record provides evidence that the inertial-based mapping system plotted the physical surface features without being disrupted by the complex lighting and visual artifacts present during data collection.

### 3.3. Predictions over Variable Speed

To evaluate whether classification confidence is dependent on vehicle velocity, prediction performance was analyzed across four distinct speed brackets (5–25 km/h).

**Figure 11:**
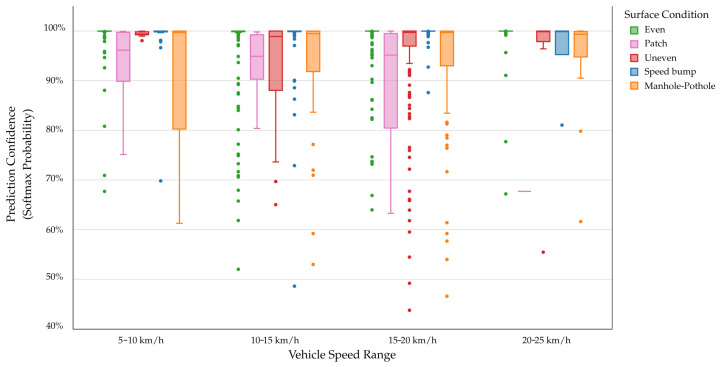
Distribution of inference confidence across four vehicle speed brackets for each target road surface condition.

Figure 11 presents the distribution of prediction confidence (softmax probability) for correct inferences across the tested speed ranges. The median prediction confidence exceeded 90% across the tested velocity spectrum for the majority of surface conditions. The Even road and Speed bump classes exhibited high stability, suggesting that the 1D-CNN+LSTM model isolates the inertial signatures of these features regardless of vehicle speed. Physical variance was observed in the Manhole-Pothole and Patch categories, where the interquartile range of confidence expanded at lower velocities.

## 4. Discussion

### 4.1. Real-Time Viability and Edge Architecture

The primary objective of this cyber-physical deployment was to establish the operational latency of the classification model and the overall architecture. The proposed edge computing architecture operated within the 11.6 ms limit required by the 86 Hz sensor polling rate. With an average total pipeline latency of 4.05 ms, the system processes inertial data with a temporal margin exceeding 60%. This result improves upon previous baseline testing [10], where the native TensorFlow model, executing on an NVIDIA GeForce RTX 4070 GPU, delivered an inference time of 33.8 ms (±1.77 ms). This indicates that the ONNX runtime is an effective strategy for optimizing deep learning models for real-time CPU execution.

To contextualize this latency, Table 3 evaluates the proposed system against alternative edge-deployment solutions. While standard microprocessor boards (e.g., Raspberry Pi 4) and Edge-AI accelerators (e.g., NVIDIA Jetson Nano) achieve comparable or higher latencies depending on the framework [28,29], these solutions often employ 8-bit quantization or simplified model architectures [30], which may reduce predictive capacity. By maintaining full model precision without quantization, the 1D-CNN+LSTM system demonstrates competitive performance within the evaluated framework. Nevertheless, it should be noted that these comparisons serve as contextual benchmarks rather than direct performance evaluations, as variations in dataset complexity, model architecture, and the specific nature of the classification tasks across studies prevent a direct comparison.

This temporal margin indicates that the MicroAutoBox III Embedded PC, while effective for establishing an operational baseline, provides higher computational resources than required for inference. The 4.05 ms execution cycle suggests that the 1D-CNN+LSTM model can operate on hardware with fewer CPU resources. This efficiency suggests that high-tier edge computers are not required, facilitating future implementations on cost-effective hardware. Therefore, the architecture provides a scalable solution for vehicle integration, mitigating bandwidth constraints and dependencies on cloud connectivity.

### 4.2. Classification Performance and Literature Comparison

The classification results presented in Table 2 and Figure 6 indicate that model performance is, as expected, influenced by the physical characteristics of the road anomalies.

The architecture demonstrates high reliability in identifying longer, extended features. The Even class achieved a 95.1% True Positive Rate (TPR), while the Speed bump class reached an 86.8% TPR. The primary misclassification for Speed bumps involved confusion with Patches (8.6%), likely because raised asphalt patches generate vibration patterns comparable to the geometric profile of a speed bump.

The Manhole-Pothole class recorded a 72.3% TPR. Misclassifications for this category were distributed between Uneven (10.1%) and Speed bump (9.7%) classes. This reflects an inherent limitation of vibration-based classification: shallow potholes generate vibrations similar to general surface roughness (Uneven), whereas severe manhole impacts produce vertical acceleration profiles that the network occasionally misidentifies as raised surface anomalies (Speed bump).

For the Patch class, the system achieved a TPR of 82.6%, with 10.9% of instances misclassified as Even. Recently installed patches often exhibit smoother profiles than the surrounding degraded pavement. In the absence of a distinct structural edge, these patches do not trigger the characteristic vertical acceleration spike required for detection. Given that patches exhibit significant heterogeneity, the classification performance for this category is inherently variable.

Overall, these results suggest that the observed errors are not random system failures but are attributable to the physical similarities between distinct road conditions. The model effectively discriminates between surface types that exhibit unique dynamic signatures, while errors occur primarily where physical profiles overlap.

The statistical metrics presented in Table 2 outline the operational strengths and limitations of the system. The narrow 95% confidence intervals for the weighted-average F1-score [0.8590, 0.8905] and the Even road class [0.9460, 0.9663] indicate that the 1D-CNN+LSTM model remains stable under typical operating conditions.

Contrarily, the Patch class exhibits a wider confidence interval [0.3815, 0.5732], reflecting the high physical variability of these features. Unlike Speed bumps or Manhole-Potholes, which possess distinct structural edges, Patches exhibit significant variance in depth, material composition, and surface wear, resulting in inconsistent inertial signatures. While the system reliably detects defined structural impacts, identifying physically heterogeneous anomalies such as Patches may require hybrid sensor fusion to improve classification consistency.

In a previous study detailing the foundational development of the classification model [10], the offline 1D-CNN+LSTM model achieved a macro-average F1-score of 0.9338. To account for how the proposed cyber-physical edge deployment affects this baseline 1D-CNN+LSTM model, a comparative analysis was conducted. Table 4 demonstrates the class-by-class predictive performance of the real-time edge architecture against the offline baseline, as well as against existing literature standards. These standards include multi-class feature engineering approaches (e.g., Raslan et al. [11]) and specialized anomaly detection algorithms using traditional machine learning and sensor fusion (e.g., Hijji et al. [31]; Celaya-Padilla et al. [32]; Shtayat et al. [33]).

As detailed in Table 4, the edge deployment maintains the detection accuracy for structurally defined features. The system recorded an F1-score of 0.9565 for Even Roads, performing similarly to the offline baseline, and achieved an F1-score of 0.8321 for Speed Bumps. This metric is consistent with specialized offline algorithms trained explicitly to detect speed bumps [32]. Consequently, the performance variance observed in this real-world deployment (Macro-average F1: 0.7756; Weighted-average F1: 0.8751) is primarily caused by the Patch class. Unlike features with defined boundaries, patches exhibit irregular depths and lack consistent geometric edges, which generate highly variable vibration patterns. Moving to a new test vehicle with different suspension dynamics physically attenuates the subtle vibration signals produced by these minor road repairs. These results establish an empirical baseline for real-time edge deployment. Future iterations will explore domain adaptation techniques to decouple the model from vehicle-specific suspension kinematics and improve cross-platform generalization.

### 4.3. Environmental Robustness

Spatial mapping validation provided evidence for the operational stability of the system under complex environmental conditions. As shown in the dashboard footage (Figure 10), the route encompassed sections characterized by irregular shadow patterns and dynamic lighting transitions. The inertial pipeline maintained continuous mapping of the physical road geometry, demonstrating robustness against optical occlusions and environmental lighting variations under the tested conditions.

### 4.4. Study Limitations

This study presents four primary limitations inherent to the proposed methodology. First, as a vibration-based architecture, detection requires direct physical contact between the vehicle tires and the road surface anomaly. Therefore, the system is restricted to mapping a single lane per vehicle pass, and the current spatial validation is constrained to a specific urban environment. While the selected test path provided sufficient diversity to capture robust samples of all five target classes, future work should include broader geographic testing across varied urban networks.

Second, system evaluation was restricted to a speed range of 5.0–25.0 km/h, consistent with the operational boundaries of the original training data. Because the 1D-CNN+LSTM model relies on fixed time-series windows, higher velocities compress the physical anomaly’s vibrational signature into fewer data points, potentially reducing the feature extraction capability. Extending functionality to highway speeds will require methodological adjustments to the time-windowing strategy.

Third, manual video annotation introduces potential temporal synchronization uncertainties. Future iterations should incorporate automated computer vision labeling pipelines or multi-expert-annotated validation protocols to establish a higher-confidence ground truth.

Finally, the empirical validation depends on a single test vehicle, meaning the inertial response is coupled to the specific suspension kinematics of that platform. Deploying the system on different vehicles requires active domain adaptation. Implementing a federated learning approach across a multi-vehicle fleet represents a viable way to recalibrate the model against diverse suspension dynamics without the need for centralizing raw inertial data.

## 5. Conclusions

This study presented an edge computing architecture for the real-time classification of road surface conditions using inertial sensors and a 1D-CNN+LSTM model. Deployed within a ROS 2 framework on a MicroAutoBox III Embedded PC, the system demonstrated consistent operational execution. By dynamically preprocessing inertial signals via mean subtraction and variance scaling, the inference node achieved an average total pipeline latency of 4.05 ms, operating within the 11.6 ms threshold established by the 86 Hz sensor polling rate, without the need for cloud connectivity.

Empirical validation across a 3.75 km urban test route produced a weighted-average F1-score of 0.8751 across five distinct road surface categories. The pipeline maintained consistent classification performance across a velocity range of 5.0–25.0 km/h. Geospatial mapping demonstrated the system’s capacity to effectively localize both continuous degradation and isolated geometric anomalies. Furthermore, the inertial-based architecture demonstrated robustness against complex lighting transitions and optical occlusions, which affect vision-based classification models.

Future research will focus on three primary objectives to expand the system’s operational feasibility. First, data collection protocols will be extended to highway velocities to evaluate the normalization pipeline against high-frequency chassis dynamics. Second, multi-vehicle validation and federated domain adaptation techniques will be explored to decouple the inference model from the suspension kinematics of a single test vehicle, enabling the development of a cross-platform architecture. Finally, future work will explore integrating the edge inference node into active vehicle monitoring loops. By employing millisecond-level classifications to inform Advanced Driver Assistance Systems, dynamic suspension tuning, and large-scale IoT infrastructure analytics, this architecture demonstrates the potential for application in next-generation ITS.

## Figures and Tables

**Table 1:** Statistical analysis of execution latency on the MicroAutoBox III Embedded PC.

Statistic	CPU Latency (ms)	Pipeline Latency (ms)
Mean	3.332	4.059
Standard Deviation	0.517	0.561
Maximum	6.23	7.14
Minimum	1.32	1.572

**Table 2:** Classification report for the real-time edge inference node.

Class	Precision	Recall	F1-Score	Support	95% CI (F1-Score)
Even	0.9618	0.9512	0.9565	820	[0.9460–0.9663]
Uneven	0.8459	0.7789	0.8110	303	[0.7744–0.8439]
Manhole-Pothole	0.8894	0.7228	0.7975	267	[0.7550–0.8356]
Patch	0.3393	0.8261	0.4810	46	[0.3815–0.5732]
Speed bump	0.7991	0.8680	0.8321	197	[0.7901–0.8702]
Accuracy	–		0.8683	1633	[0.8512–0.8849]
Macro Avg	0.7671	0.8294	0.7756	1633	[0.7477–0.8022]
Weighted Avg	0.8913	0.8683	0.8751	1633	[0.8590–0.8905]

**Table 3:** Contextual benchmarking of computational latency across various processing platforms.

Hardware Platform	Deployment Context	Model Precision	Latency (ms)
Raspberry Pi 4 [28,29]	Multimodal/Sensor Fusion	Quantized/8-bit	45.0–230.0
NVIDIA Jetson Nano [28,29]	Time-series/TensorRT	Quantized/Optimized	12.0–116.0
GeForce RTX 4070 [10]	Offline Baseline (TensorFlow)	Full Precision	33.8
MicroAutoBox III (Proposed)	Real-Time Edge (ONNX)	Full Precision	4.05

**Table 4:** Class-by-class F1-score comparison of the proposed edge architecture against offline baseline and literature standards.

Target Condition	This Work	Arce-Saenz et al. [10]	Raslan et al. [11]	Specialized/Binary
Even Road	0.9565	0.9495	0.9569	0.97 ^a^
Uneven Road	0.811	0.8922	0.9053	–
Manhole/Pothole	0.7975	0.8866	0.9476	0.899 ^b^
Patch	0.481	0.951	–	0.96 ^a^
Speed Bump	0.8321	0.99	0.925	0.9355 ^c^
Macro-Avg	0.7756	0.9338	0.9337	–

^a^ Shtayat et al. [33] (Traditional machine learning models). ^b^ Hijji et al. [31] (Binary CNN Fusion, Potholes). ^c^ Celaya-Padilla et al. [32] (Genetic Algorithm, Speed bumps).

## Data Availability

The data presented in this study are available on request from the corresponding author.

## Abbreviations

The following abbreviations are used in this manuscript:

AI	Artificial Intelligence
CNN	Convolutional Neural Network
GPS	Global Positioning System
IMU	Inertial Measurement Unit
ITS	Intelligent Transportation Systems
LiDAR	Light Detection and Ranging
LSTM	Long Short-Term Memory
ONNX	Open Neural Network Exchange
ROS 2	Robot Operating System 2
TRP	True Positive Rate
